# Anti-Salmonella Activity of a Novel Peptide, KGGDLGLFEPTL, Derived from Egg Yolk Hydrolysate

**DOI:** 10.3390/antibiotics13010019

**Published:** 2023-12-24

**Authors:** Thippawan Pimchan, Fu Tian, Kanjana Thumanu, Sureelak Rodtong, Jirawat Yongsawatdigul

**Affiliations:** 1 School of Food Technology, Institute of Agricultural Technology, Suranaree University of Technology, Nakhon Ratchasima 30000, Thailand; 565258@g.sut.ac.th; 2 College of Food and Pharmaceutical Engineering, Guizhou Institute of Technology, Guiyang 550003, China; tianfu@git.edu.cn; 3 Synchrotron Light Research Institute (Public Organization), Nakhon Ratchasima 30000, Thailand; kanjanat@slri.or.th; 4 School of Preclinical Sciences, Institute of Science, Suranaree University of Technology, Nakhon Ratchasima 30000, Thailand; sureelak@sut.ac.th

**Keywords:** egg yolk protein, antimicrobial peptide, food-borne pathogens, *Salmonella* Typhimurium

## Abstract

The present study aimed to characterize the mode of action of a novel antimicrobial peptide isolated from egg yolk hydrolysate. The EYHp6, KGGDLGLFEPTL, exhibited inhibition against *Salmonella enterica* serovar Typhimurium TISTR 292 and *S. enterica* serovar Enteritidis DMST 15679 with a MIC value of 2 mM. In contrast, *S*. *enterica* serovar Newport ATCC 6962 and other strains of Typhimurium and Enteritidis were inhibited at 4 mM. EYHp6 increased the cell membrane permeability of *S*. Typhimurium TISTR 292, leading to DNA leakage. Membrane integrity determined by propidium iodide and SYTO9 staining visualized by confocal microscopy demonstrated that EYHp6 at 1 × MIC induced disruption of cell membranes. Electron microscopy revealed that treatment of *S.* Typhimurium with EYHp6 led to damage to the cell membrane, causing the leakage of intracellular contents. Synchrotron-based Fourier-transform infrared spectroscopy indicated that EYHp6 killed *S.* Typhimurium by targeting fatty acids and nucleic acids in the cell membrane. The peptide did not show hemolytic activity up to 4 mM. These findings suggest that EYHp6 could be a promising antibacterial agent for controlling the growth of *S*. *enterica*.

## 1. Introduction

Contaminations of animal-based food products during production and retailing processes with foodborne pathogenic microorganisms are of serious concern. According to the World Health Organization (WHO), unsafe food can cause up to 600 million foodborne illness cases, and 420,000 people die each year, resulting in productivity loss and significant medical costs in low- and middle-income countries [1]. *Salmonella* spp. is the third leading cause of death among foodborne outbreaks worldwide [2]. Approximately 93 million gastroenteritis cases and 155,000 deaths caused by non-typhoidal *Salmonella* are reported annually worldwide [3]. *S*. *enterica* serovar Enteritidis is one of two species of *Salmonella* spp., comprising six subspecies with approximately 2659 serovars [4]. Serovars can trigger infections in both humans and animals, including Enteritidis and Typhimurium, which are mainly transmitted by animal-based foods, such as beef, pork, poultry, and raw eggs [5,6]. The use of antibiotics is one of the common measures for pathogenic bacteria control. However, various negative side effects of antibiotics have been reported, including the development of drug resistance, transfer of antibiotic-resistant bacteria to humans, and hyper sensitivity reaction. Some antibiotics, namely sulfamethazine, oxytetracycline, and furazolidone, have been reported to have carcinogenicity, while chloramphenicol induces bone marrow toxicity. In addition, disturbance of gut microbiota by tetracyclines is reported [7,8]. Natural antimicrobial agents that do not cause drug resistance and are safe for consumers should be sought. 

Antimicrobial peptides (AMPs) are small peptides that play an important role in host defense from microbial invasion [9]. Since AMPs have broad-spectrum activity and find it difficult to develop resistance, they have been receiving increasing attention as alternative antibiotics. Most AMPs, predominantly cationic peptides, comprise less than 50 amino acids [10], which can electrostatically interact with anionic components of bacterial membranes [11]. AMPs disrupt the integrity or function of phospholipid bilayers of cell membranes directly through toroidal, carpet, aggregate, or barrel models [12]. Thus, AMPs have a lower possibility of inducing drug resistance. In contrast, specific receptors and/or targets of antibiotics can be modified to develop resistance [13]. A number of AMPs have been found to have good antimicrobial potential and are applicable as food preservatives, such as HX-12C (FFRKVLKLIRKIWR), HSEP3 (RSVIFGCTKSIPPICFVGFK), LCWAP (FTKPGVCPRRRWGAG), brevilaterins, and various bacteriocins [14,15,16,17,18]. In addition, the exploration of new bioactive peptides from enzymatic hydrolysis of protein is a great avenue for alternative AMPs [19]. Enzymatic hydrolysis is well-established as a rapid and robust method for the production of bioactive peptides [20]. It has been recently found that enzymatic hydrolysis of certain proteins results in peptides that exhibit antimicrobial activity [21]. Several AMPs have been isolated from hydrolysates of various protein sources [22,23,24].

Egg yolk proteins are the main coproduct obtained from lecithin extraction. Their biological and biotechnological value is reduced by extraction solvent [25]. In our previous study, egg yolk proteins hydrolyzed by pepsin exhibited antimicrobial activity against *Staphylococcus aureus* ATCC 29213 and *S.* serovar Typhimurium TISTR 292 when fractionated on C-18 column, with a feasible mechanism targeting the cell membranes. The most potent peptide responsible for such activity has been identified to be EYHp6 (KGGDLGLFEPTL), and it showed potent antimicrobial activity against *S.* Typhimurium [26]. Therefore, EYHp6 is a good candidate for further development as a new antimicrobial agent. However, its mode of action against *S.* Typhimurium is unknown, which would limit further application. Thus, the objective of this study was to unveil the antimicrobial mechanism of EYHp6 against *S.* Typhimurium. Moreover, synchrotron radiation-based Fourier transform infrared microspectroscopy (SR-FTIR) was applied to evaluate the cellular changes induced by EYHp6. The toxicity of peptides was also determined by evaluating the hemolytic activity against human red blood cells.

## 2. Results and Discussion

### 2.1. Antimicrobial Activity

The antimicrobial activities of peptide EYHp6 were evaluated against various serovars of *S*. *enterica*, including *S*. Typhimurium TISTR 292, *S*. Typhimurium ATCC 14028, *S*. Enteritidis DMST 15679, *S*. Enteritidis ATCC 13076, and *S*. serovar Newport ATCC 6962. The MIC values are shown in Table 1. Peptide EYHp6 showed anti-salmonella activity with MIC values ranging from 2 to 4 mM, depending on serovar. The peptide was more effective against *S*. Typhimurium TISTR 292 and *S*. Enteritidis DMST 15679 with a MIC value of 2 mM. *S*. Enteritidis and *S*. Typhimurium are among the most important serovars causing enteric infections in various animals and humans [4]. The peptide also showed antibacterial effects against *S*. Newport, although with a higher MIC value. *S*. Newport is considered an emerging multidrug-resistant serovar, including ampicillin, chloramphenicol, streptomycin, sulfamethoxazole, and tetracycline, which causes severe infections and death in animals and humans [27].

EYHp6, KGGDLGLFEPTL, revealed antibacterial activity with MIC at 2 mM against *S.* Typhimurium TISTR 292, which was consistent with our previous study [26]. EYHp6 did not match with any peptides in the BIOPEP-UWM, NCBI, and APD databases, suggesting it is a novel AMP. EYHp6 contained 12 amino acids with 45% hydrophobicity and a net charge of −1. The MIC value of EYHp6 was comparable to those of anionic antimicrobial peptides AP1 (GEQGALAQFGEWL) and MOp3 (MCNDCGA) [28,29] and cationic antimicrobial peptide BCp12 (YLGYLEQLLRLK) [30], which ranged from 1 to 4.4 mM. Peptides derived from protein hydrolysate usually exhibit MIC values in mM levels, which are less potent than those derived from natural sources whose MIC values typically range in µM levels [21,31,32]. The antibacterial activity of peptides depends on several factors, including net charge, secondary structure, hydrophobicity, and amino acid sequences [31,33]. The antibacterial activity of the EYHp6 peptide could be attributed to its anionic characteristic (net charge − 1). Anionic AMP is thought to induce membrane destabilization by interacting with membrane lipids and/or changing the hydrophobicity of the cell surface, leading to the alteration of cell membrane permeability [34]. In addition, some anionic AMPs can form a cationic salt bridge via metal ions like Zn^2+^, allowing interactions with negatively charged bacterial membranes. This would ultimately weaken the cell membrane and increase its permeability [35]. The majority of AMPs are cationic in nature, binding to the negatively charged bacterial membrane, increasing membrane permeability and subsequently leading to cell lysis and leakage of intracellular components. Additionally, the amphiphilicity of most AMPs can facilitate the integration of AMPs into the lipid bilayers of the membrane, causing the disintegration of the cell membrane and cell death [36]. Few anionic AMPs have been reported. Zhao et al. [37] reported that anionic AMP, HVLDTPLL, isolated from hydrolysates of *Moringa oleifera* seed proteins, displayed activity against *S.* Typhimurium CICC 21484 at 4.4 mM RVAPEEHPTL and FFTQATDLLSR, which were anionic AMPs derived from Maillard reaction products and inhibited the growth of *Escherichia coli* at approximately 17 and 15 mM, respectively [38]. These reported peptides showed less effective antimicrobial activity than the EYHp6 reported in our study. 

### 2.2. Time Kill Kinetics

**Figure 1:**
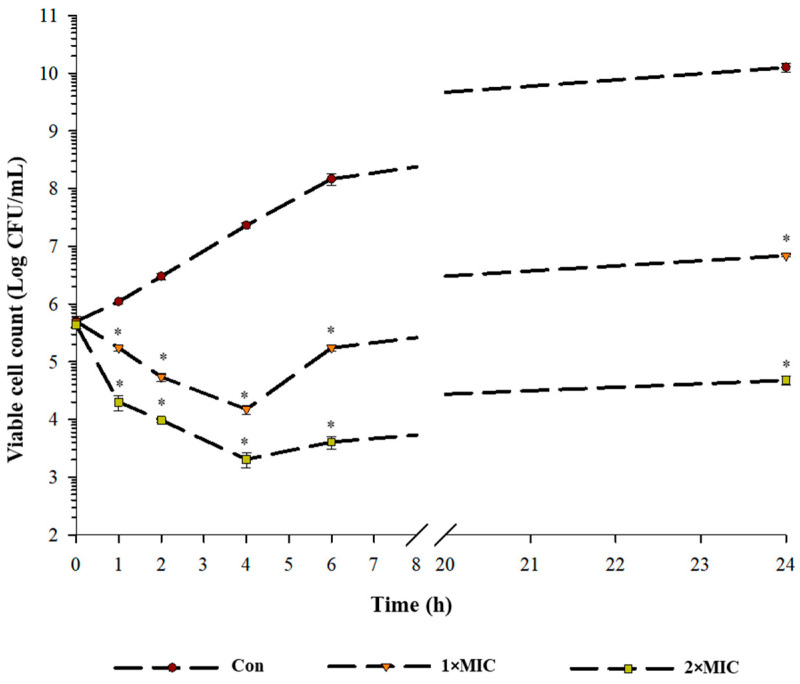
Time-killing kinetics of EYHp6 against *S.* Typhimurium TISTR 292 at 1 × MIC and 2 × MIC concentrations. All data are expressed as the mean values of triplicate ± standard deviation. * indicates a significant difference (*p* < 0.05) compared with control at each time interval.

The time-kill kinetic curves of *S*. Typhimurium TISTR 292 in the presence and absence of EYHp6 are shown in Figure 1. In the control (no peptide), bacteria grew continuously over the period of 24 h. In the presence of 1 × MIC peptide, cell viability was reduced by approximately 1 Log CFU/mL after 4 h of incubation compared to the initial inoculum. Subsequently, growth was resumed, reaching 6 Log CFU/mL at 24 h. The final cell count at 24 h was approximately 4 Log CFU/mL lower than that of the control. During the 2 × MIC treatment, cell count decreased to a greater extent than that of the 1 × MIC treatment. After 24 h incubation, the 2 × MIC treatment showed approximately 2 Log CFU/mL reduction compared to the initial count. It should be noted that the cell viability of the 2 × MIC treatment was approximately 6 Log CFU/mL lower than the control (*p* < 0.05). These results demonstrated that the growth of *S*. Typhimurium TISTR292 can be controlled by EYHp6 at 2 × MIC.

### 2.3. CD Spectroscopy

**Figure 2:**
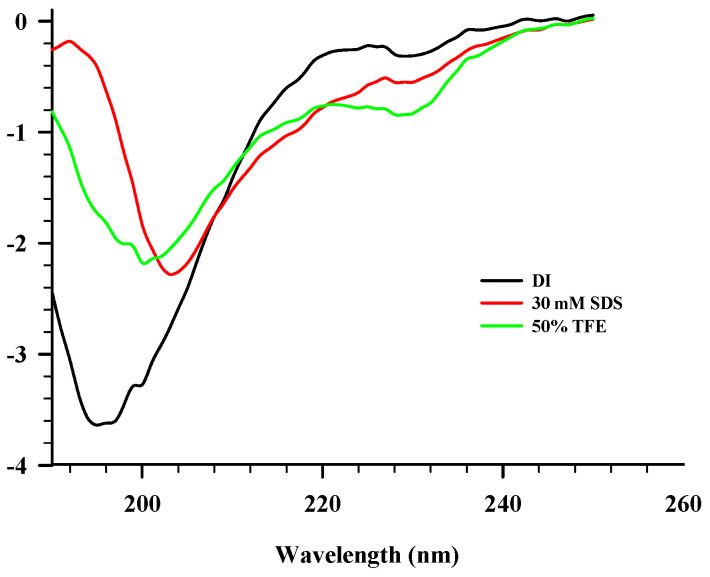
CD spectra of EYHp6 in various solvents mimicking cell membrane environments.

The CD spectra of EYH6 in various solvents are shown in Figure 2. The secondary structure of the peptide in aqueous solution exhibited a random coil structure with a negative peak around 200 nm. In 50% TFE and 30 mM SDS (mimicking negatively charged prokaryotic membrane), the CD spectra of EYH6 showed minor alteration in the secondary structure with predominant random coil structures. Although typical structures adopted by AMPs in contact with membranes are α-helical and β-sheet, AMPs with random coil structures have also been reported [39]. Souza et al. [40] showed that two AMPs, PepGAT (GATIRAVNSR) and PepKAA (KAANRIKYFQ), displayed a random chain conformation in aqueous solution, organic solvent (50% TFE), and upon binding to negatively charged lipid systems. Indolicidin, a small cationic AMP with 13 amino acid residues, was reported to possess a broad spectrum against Gram-negative and -positive bacteria, protozoa, fungi, and viruses. A short chain of indolicidin confers it largely linear in structure, forming random coil structures in solution [41]. Peptides with a random coil structure are capable of inserting themselves into the outer leaflet of the model lipid bilayer [42]. Our study suggested that EYHp6 exhibited a random coil structure when interacting with lipid components of the cytoplasmic membrane.

### 2.4. Membrane Disruption

**Figure 3:**
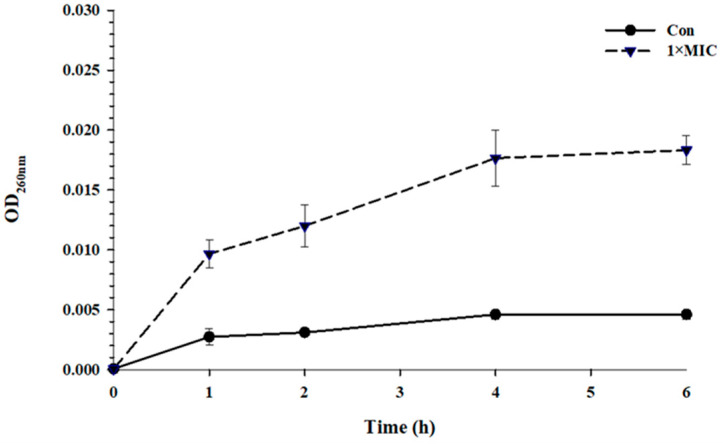
Release of nucleic acids from *S.* Typhimurium TISTR 292 treated with 1 × MIC of EYHp6. All data are expressed as the mean values of triplicate ± standard deviation.

An increase in OD_260_ was observed when *S.* Typhimurium was incubated with EYHp6 at 2 mM for 1 h (Figure 3), indicating leakage of genetic materials. A substantial increase in absorbance was evident at 4 h of peptide exposure, implying an increase in membrane permeability. Zhou et al. [43] reported a similar finding, in which the leakage of intracellular nucleic acid and protein in *E. coli* increased when exposed to peptide LL-1 isolated from *Dichocrocis punctiferalis*. Longer exposure time (>4 h) did not further enhance cell membrane leakage. This could probably be due to the recovery of injured cells, as evidenced by cell growth after 4 h of exposure time (Figure 1). 

**Figure 4:**
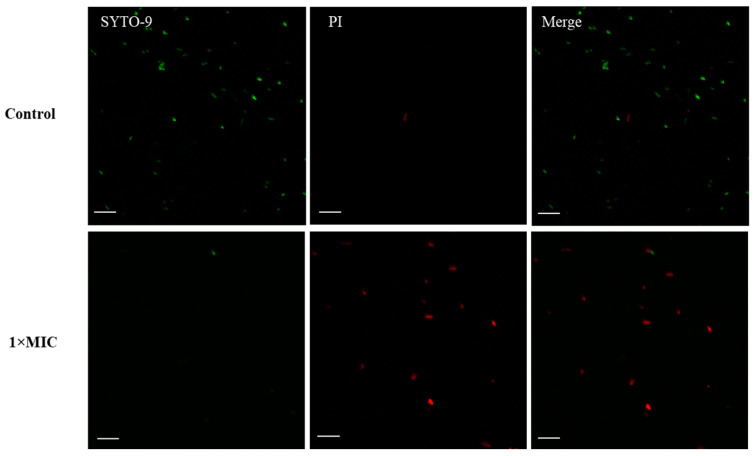
Confocal fluorescence microscopic images of *S*. Typhimurium incubated with EYHp6 at 1 × MIC and the control (without peptide). All samples were stained by SYTO-9 and PI. Bar = 10 µm and magnification of 60×.

PI and SYTO 9 are widely used to elucidate cell membrane permeability. SYTO 9 can pass through living and dead cell membranes and bind DNA and RNA to produce green fluorescence. Meanwhile, PI can penetrate dead cells or cells with damaged membranes and bind to DNA, emitting a red fluorescence signal [44]. Most untreated cells (control) showed only a green fluorescent signal (Figure 4), demonstrating the integrity of cell membranes. In contrast, red fluorescence signals were detected in *S.* Typhimurium treated with 2 mM EYHp6 for 4 h. Song et al. [45] found that AMP (KDFPGRR) increased the membrane permeability of *E. coli*, resulting in an increase in PI-signal after exposure to the peptide at 0.5 mg/mL for 30 min. Hou et al. [46] reported that more than 57.3% of cells were stained with PI after exposure to the peptide Cp1 (LRLKKYKVPQL) derived from bovine αS1-casein hydrolysate. Our results indicate that EYHp6 induced disruption of the cell membrane of *S.* Typhimurium. 

### 2.5. Electron Microscopy

**Figure 5:**
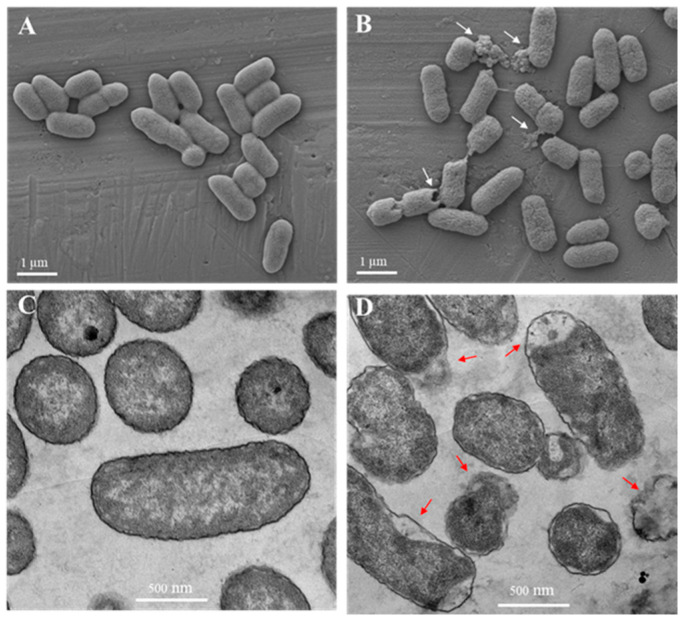
Scanning (**A**,**B**) and transmission electron (**C**,**D**) microscopy images of *S*. Typhimurium. (**A**,**C**) Control sample without peptides, and (**B**,**D**) cells treated with EHYp6 at 1 × MIC for 4 h (**B**,**D**). White and red arrows represent the observed morphological changes.

In the absence of EYHp6, *S.* Typhimurium TISTR 292 exhibited intact cells with smooth surfaces (Figure 5A). Cell damage was observed with an irregular cell surface, pore formation, and cell lysis after the bacterial cells were exposed to EYHp6 at 1 × MIC for 4 h (Figure 5B). In addition, TEM results illustrated that untreated cells showed intact cell membranes and were full of intracellular contents, while EYHp6-treated cells demonstrated the collapse of the cytoplasmic membrane with wider periplasmic space (Figure 5C,D). In addition, leakage of intracellular materials was also observed. 

SEM and TEM images showed that EYHp6 inactivated *S*. Typhimurium by disrupting the cell membrane, leading to the dissolution of the cytoplasmic space and leakage of intracellular contents. The peptide CCCPKAF, isolated from chicken plasma, induced the death of *Bacillus cereus* by forming pores and damaging cell membranes [47]. The amphiphilic structure of anionic AMPs is believed to play a key role in bacterial membrane interactions [48]. 

### 2.6. SR–FTIR Microspectroscopy

**Figure 6:**
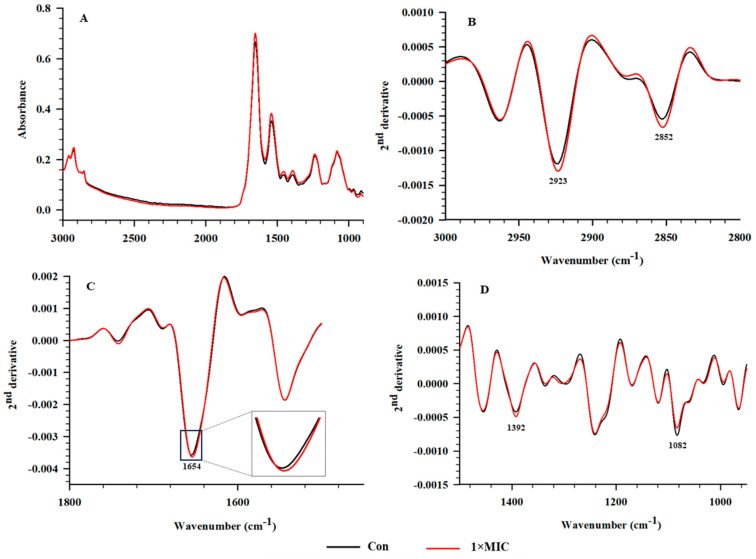
Average original SR–FTIR spectra and average the second derivative spectra of *S*. Typhimurium without peptide treatment (control) and cells treated with 1 × MIC EYHp6. (**A**) Average original FTIR spectra (3800–900 cm^−1^), (**B**) average second derivative spectra of fatty acid regions (3000–2800 cm^−1^), (**C**) protein (1700–1500 cm^−1^), and (**D**) mixed regions of fatty acids of fatty acids, proteins and phosphate–carrying molecules (1500–1200 cm^−1^), and nucleic acid and other carbohydrate regions (1200–950 cm^−1^). Triplicate experiments were conducted, and a total of 150 spectra were averaged.

SR–FTIR microspectroscopy, providing much higher brightness and smaller detection spots than conventional FTIR, was employed to evaluate the alteration of cellular components of *S*. Typhimurium treated with EYHp6 (Figure 6A). SR–FTIR spectra can be divided into four regions, namely 3000–2800 (fatty acids in the bacterial cell membrane), 1700–1500 (proteins and peptides), 1500–1200 (the mixed regions of fatty acids, proteins and phosphate–containing molecules), and 1200–900 cm^−1^ (polysaccharides as well as nucleic acids) [49]. The second derivatives of spectra indicated that cells treated with 2 mM EYHp6 exhibited distinct changes in the fatty acid regions at 2923 and 2852 cm^−1^ and nucleic acid regions at 1083 cm^−1^ compared to the control (Figure 6B,D). Slight changes around the amide I of protein were also observed (Figure 6C). Asymmetrical vibrations of –CH groups of fatty acids on the membranes and asymmetric stretching of phosphate group P=O of the phosphodiester bond of nucleic acids indicated that EYHp6 induced structural changes in lipid membranes and genetic materials of *S*. Typhimurium. A slight effect on intracellular proteins could also take place. 

**Figure 7:**
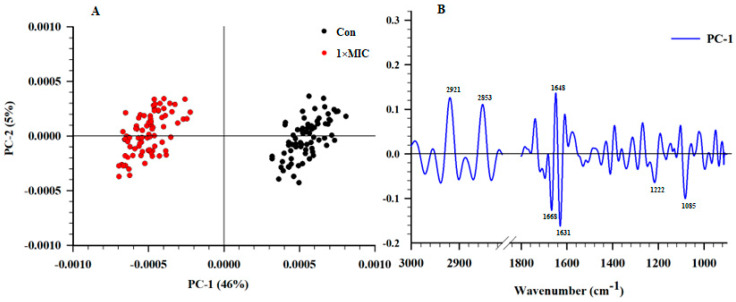
PCA analysis of the SR-FTIR spectra of *S*. Typhimurium without peptides (control) and cells incubated with 1 × MIC for 4 h. (**A**) PCA of 2D score plot and (**B**) PCA loading plot.

The two–dimensional PCA plot demonstrated that spectra of the untreated control and cells treated with 1 × MIC of EYHp6 were explicitly separated along PC1 with a total variation of 46% (Figure 7A). The PC1 loading plots showed the highest positive spectrum variation at 2921, 2853, and 1648 cm^−1^ (Figure 7B), indicating changes in fatty acids and proteins in cells treated with EYHp6. In contrast, the negative loading at 1668, 1631, 1222, and 1085 cm^−1^ is attributed to C=O stretching of amide groups of proteins and P=O symmetric stretching from phosphodiester bond of cellular nucleic acids. These results indicate that EYHp6 induced modification of proteins and nucleic acids of *S*. Typhimurium. The antimicrobial peptide (KVFLGLK) from *Jatropha curcas* inhibits the growth of *S. aureus* ATCC 25923 by disrupting the cell wall and cell membrane, as evidenced by FT–IR spectra of proteins, fatty acids, polysaccharides, and the global glucan portions [50]. Based on these SR–FTIR results, EYHp6 may primarily affect membrane lipids of the *S*. Typhimurium and induce changes in nucleic acids and intracellular proteins, leading to cellular damage and death.

### 2.7. Hemolytic Activity

**Figure 8:**
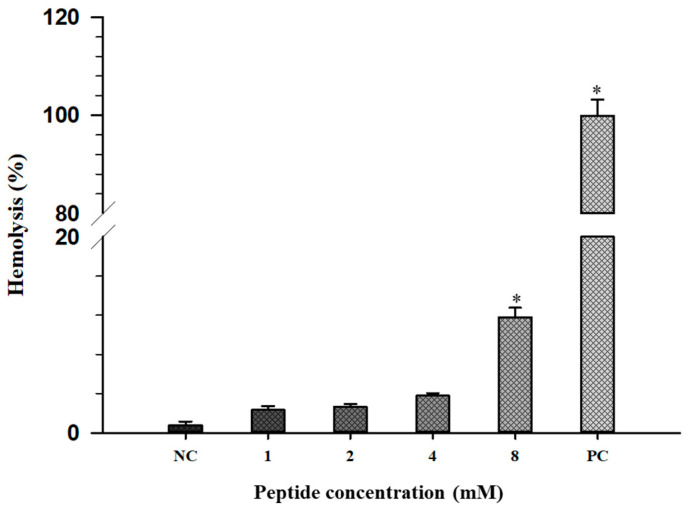
Hemolytic activity in human red blood cells of EHYp6. Data are average of at least 4 independent experiments. Error bars represent the standard deviations. * *p* < 0.05 compared to the negative control group. PBS and 10% Triton–X–100 were used as negative (NC) and positive controls (PC).

The hemolytic activity of EHYp6 on human erythrocytes was used to reflect the cytotoxic effect of the peptide on mammalian cells. The hemolysis rate of EYHp6 was increased with various concentrations (Figure 8). A hemolysis rate below 5% is generally regarded as safe for red blood cell integrity [51]. Complete hemolysis of RBC was observed in the presence of 0.1% Triton X–100, while PBS had no effect (Figure 8). It was reported that BCp12 peptide derived from buffalo casein hydrolysate did not disrupt the RBC of rabbit erythrocytes at 1.3 mM [30]. The SP–1 peptide, KLVDASHRLATGDVAVRA, from protein hydrolysates of *Spirulina platensis* exhibited no hemolytic activity up to a concentration of 8 × MIC values at 68.1 mM [52]. In contrast, melittin, a well–known hemolytic peptide, showed 50% hemolysis in rabbit and human erythrocytes at only 2.6 μM [53]. Our results suggested that EYHp6 did not show a hemolytic effect at 4 mM, which was two times higher than the MIC value. This finding implies that EYHp6 is likely safe for food application.

## 3. Materials and Methods

### 3.1. Peptide Synthesis

EYHp6 (KGGDLGLFEPTL), identified by Pimchan et al. [26], was synthesized using a solid–phase peptide synthesis method (GL Biochem Ltd., Shanghai, China). It was purified using an HPLC to achieve 95% purity. Liquid chromatography equipped with a mass spectrometer (LC–MS/ESI) was employed to verify the molecular mass of the synthetic peptide. 

### 3.2. Antimicrobial Activity Assay 

The antibacterial activity of synthetic peptides against *S.* Typhimurium TISTR 292, *S.* Typhimurium ATCC 14028, *S.* Enteritidis DMST 15679, *S.* Enteritidis ATCC 13076, and *S.* Newport ATCC 6962 was evaluated by the broth microdilution method following the standard guideline described by the Clinical Laboratory Standard Guideline (CLSI) with some modifications. All strains were cultured on tryptic soy agar and incubated at 37 °C for 18–24 h. Bacterial cells were then diluted to 10^5^ CFU/mL with Mueller–Hinton broth. In total, 50 µL of cell suspension and 50 μL of peptide at various concentrations were added to 96–well plates and then incubated for 18 h at 37 °C. Bacterial cells without peptides served as a negative control. Inhibition of bacterial growth was determined at 600 nm using a microplate reader (Varioskan LUX, Thermo Scientific, Vantaa, Finland). The minimum inhibitory concentration (MIC) was defined as the lowest concentration of peptide that completely inhibits the growth of bacteria.

### 3.3. Time Killing Assay

The kinetic inhibition of EYH6 against *S*. Typhimurium TISTR 292 was determined to confirm its antibacterial action. Briefly, overnight–grown culture was diluted with MH broth to obtain a cell density of 10^5^ CFU/mL, followed by adding EYH6 at concentrations of 1 × MIC and 2 × MIC. In total, 100 µL of cell suspension were taken from different exposure time intervals (0, 1, 2, 4, 6, 8, and 24 h) at 37 °C and diluted with normal saline before dropping 10 µL on a Mueller–Hinton agar plate and incubated at 37 °C overnight. The growing colonies were counted and expressed as CFU/mL.

### 3.4. Circular Dichroism (CD) Analysis

The secondary structure of EYH6 in different solutions was determined by CD, according to Liu et al. [54]. The peptide was solubilized in DI water representing an aqueous environment, 30 mM sodium dodecyl sulfate (SDS) simulating negatively charged membranes, or 50% trifluoroethanol (TFE) mimicking hydrophobic conditions in the cell membrane. The CD of EYH6 at 0.2 mg/mL was measured using a Jasco J–815 Spectropolarimeter (Jasco, Tokyo, Japan) set at 25 °C. CD spectra at 190–250 nm were recorded using a quartz dish with a diameter of 1 mm, a slit of 2 nm, and a scanning speed of 50 nm/min. An analysis of acquired CD spectra was performed using the BestSel server (http://bestsel.elte.hu/index.php (accessed on 19 May 2023)) to evaluate the secondary structures of the peptide.

### 3.5. Leakage of Nucleic Acids

Leakage analysis of nucleic acids of *S.* Typhimurium TISTR 292 treated with peptide was conducted following the method of Tian et al. [47] with minor modifications. Bacterial suspensions were incubated with 1 × MIC EYHp6 37 °C for 4 h. The bacterial suspension without peptide was used as a control. At 1 h intervals, samples were taken for centrifugation at 10,000× *g* for 5 min, and supernatants were collected and filtered through a 0.22–µm syringe filter. Nucleic acid contents were estimated at 260 nm using NanoDrop™ 2000c spectrophotometers (Thermo Scientific, Waltham, MA, USA).

### 3.6. Electron Microscopy

The morphology and structural changes in *S.* Typhimurium TISTR 292 treated with EYHp6 were evaluated using a scanning electron microscope (SEM) and transmission electron microscope (TEM). The mid–log grown cultures were treated with 1 × MIC of EYHp6 at 37 °C for 4 h. Cells were collected by centrifuging at 3000× *g* and 4 °C for 10 min and washed twice with sterile PBS. For SEM, samples were fixed overnight with 2.5% (*w*/*v*) glutaraldehyde, followed by 1% osmium tetroxide fixation and dehydration by acetone, as detailed by Pimchan et al. [26]. The samples were coated with carbon and gold. A field-emission scanning electron microscope (Zeiss AURIGA FESEM/FIB/EDX, Jena, Germany) was applied with electron energy between 2 and 5 keV.

Sample preparation for TEM was also carried out as described for SEM. After serial dehydration with acetone, samples were infiltrated in a mixture of absolute acetone and epoxy resin (*v*/*v*) at 1:3 and 1:1 for 3 h, followed by 3:1 overnight. Subsequently, samples were infiltrated with 100% epoxy resin and polymerized in an oven at 60 °C for 24 h in an embedding capsule. Ultrathin sections with a thickness of 70–90 nm were performed using an ultramicrotome equipped with a diamond knife. The sections were subsequently collected on 200–mesh copper grids and poststained with uranyl acetate and lead citrate for 15 min under ambient conditions. A Tecnai G2 electron microscope (FEI, Hillsboro, OR, USA) at 120 kV was applied to record micrographs. 

### 3.7. Confocal Laser Scanning Microscopy (CLSM)

Membrane integrity tests of *S.* Typhimurium TISTR 292 treated with EYHp6 were carried out following the method of Liao et al. [55]. Cells in the logarithmic growth stage at a final concentration of 10^7^ CFU/mL were challenged with the peptide at 1 × MIC for 4 h at 37 °C. The samples were centrifuged at 3000× *g* for 10 min. Cells were washed twice with PBS and stained with 5 μg/mL SYTO–9 and 10 μg/mL propidium iodide (PI) for 30 min at 4 °C in the dark. Excessive staining was removed by washing it with PBS 3 times prior to dropping it on a glass slide to view under a confocal laser scanning microscope (Nikon 90i A1R, Nikon, Tokyo, Japan). Untreated bacterial cells were set as a negative control. Excitation and emission wavelengths of 488 and 530 nm were used for SYTO–9-stained cells, while wavelengths of 538 and 617 nm, respectively, were applied for PI–stained cells.

### 3.8. Synchrotron–Fourier–Transform Infrared Spectroscopy (SR–FTIR)

Changes in cellular components of *S.* Typhimurium TISTR 292 treated with EYHp6 peptide were determined by SR–FTIR analysis, as detailed by Tian et al. [47]. Mid–logarithmic growing cells (10^8^ CFU/mL) were incubated at 37 °C for 4 h with 1 × MIC of the peptide. An IR–transparent 2 mm thick barium fluoride (BaF_2_) window was used. All spectra were obtained from transmission mode using a Vertex 70 FTIR spectrometer coupled with an IR microscope Hyperion 2000 (Bruker Optics, Ettlingen, Germany) connected to the Synchrotron Light Source at the beamline 4.1 at the Synchrotron Light Research Institute (Nakhon Ratchasima, Thailand). Data analysis was performed using OPUS 7.5 software (Bruker Optics Ltd., Ettlingen, Germany). Savitzky–Golay algorithms with seventeen smoothing points were applied to calculate the signal intensity of second derivatives. Principal component analysis (PCA) was performed using Unscramble X software version 10.4 (CAMO Software AS, Oslo, Norway). 

### 3.9. Hemolysis

The effect of synthetic peptides on hemolytic activity of erythrocytes was carried out as previously described by Stark et al. [56]. Fresh blood from healthy donors was obtained based on the protocol approved by the Human Research Ethics Committee of the Suranaree University of Technology (EC–64–32). Red blood cells (RBC) were washed three times with PBS and diluted to 1% RBC in PBS. In total, 50 µL of RBC suspension were added to 50 µL of various concentrations of peptide and incubated for 1 h at 37 °C. The RBC was mixed with PBS, and 10% Triton–X–100 was also prepared as a negative and positive control, respectively. After 5 min centrifugation at 1000× *g* (4 °C), the release of hemoglobin was measured at 570 nm using a microplate reader (Molecular Devices, Sunnyvale, CA, USA). The hemolytic activity was calculated as follows:

$$\mathrm{Hemolysis}\left(\%\right)=\frac{{\mathrm{OD}}_{570}\;\mathrm{of}\;\mathrm{the}\;\mathrm{treated}\;\mathrm{sample}-{\mathrm{OD}}_{570}\;\mathrm{of}\;\mathrm{the}\;\mathrm{negative}\;\mathrm{control}}{{\mathrm{OD}}_{570}\;\mathrm{of}\;\mathrm{the}\;\mathrm{positive}\;\mathrm{control}-{\mathrm{OD}}_{570}\;\mathrm{of}\;\mathrm{the}\;\mathrm{negative}\;\mathrm{control}}\times 100$$

### 3.10. Statistical Analysis

All experiments were performed in 3 independent replications. Results were expressed as mean ± SD. Statistical analysis was performed using SPSS version 23.0 software (SPSS Inc., Chicago, IL, USA), with one–way analysis of variance (one–way ANOVA) and Tukey’s HSD post hoc test. A *p* < 0.05 was regarded as statistically significant.

## 4. Conclusions

The novel EYHp6 peptide isolated from the egg yolk protein hydrolysate is an antibacterial agent against several serovars of *S. enterica*, particularly *S.* Typhimurium TISTR 292 and *S*. Enteritidis ATCC 13076. The anionic EYHp6 disrupted the cell membrane, leading to an increase in permeability and, eventually, cell death. SR–FTIR analysis revealed that EYHp6 induced alterations in the lipid membranes and nucleic acids of *S.* Typhimurium. EYHp6 had no toxicity on human red blood cells at concentrations up to 2 × MIC (4 mM). This peptide could be a good candidate for further development as a novel antibacterial agent against foodborne pathogens. Further studies on the efficacy of EYHp6 on microbial control in food samples and on gut microbiota would pave the way to the application of AMP in food and functional food products.

## Figures and Tables

**Table 1:** Antibacterial activities of peptide EYHp6 against various serovars of *S. enterica*.

Serotype	MIC (mM)
*S.* Typhimurium TISTR 292	2
*S.* Typhimurium ATCC 14028	4
*S.* Enteritidis DMST 15679	2
*S.* Enteritidis ATCC 13076	4
*S*. Newport ATCC 6962	4

## Data Availability

All data are contained within the article.

## References

[1] World Health Organization (2020). Food Safety. https://www.who.int/news-room/fact-sheets/detail/food-safety.

[2] Todd E. (2014). Foodborne diseases: Overview of biological hazards and foodborne diseases. Encycl. Food Saf..

[3] Majowicz S.E., Musto J., Scallan E., Angulo F.J., Kirk M., O’Brien S.J., Jones T.F., Fazil A., Hoekstra R.M., International Collaboration on Enteric Disease “Burden of Illness” Studies (2010). The global burden of nontyphoidal *Salmonella* gastroenteritis. Clin. Infect. Dis..

[4] Ferrari R.G., Rosario D.K., Cunha-Neto A., Mano S.B., Figueiredo E.E., Conte-Junior C.A. (2019). Worldwide epidemiology of *Salmonella* serovars in animal-based foods: A meta-analysis. Appl. Environ. Microbiol..

[5] Ehuwa O., Jaiswal A.K., Jaiswal S. (2021). *Salmonella*, food safety and food handling practices. Foods.

[6] Wessels K., Rip D., Gouws P. (2021). *Salmonella* in chicken meat: Consumption, outbreaks, characteristics, current control methods and the potential of bacteriophage use. Foods.

[7] Arsène M.M.J., Davares A.K.L., Viktorovna P.I., Andreevna S.L., Sarra S., Khelifi I., Sergueïevna D.M. (2022). The public health issue of antibiotic residues in food and feed: Causes, consequences, and potential solutions. Vet. World.

[8] Manyi-Loh C., Mamphweli S., Meyer E., Okoh A. (2018). Antibiotic use in agriculture and its consequential resistance in environmental sources: Potential public health implications. Molecules.

[9] Fox J.L. (2013). Antimicrobial peptides stage a comeback: Better understanding of the mechanisms of action, modification and synthesis of antimicrobial peptides is reigniting commercial development. Nat. Biotechnol..

[10] Rahman M.A., Bam M., Luat E., Jui M.S., Ganewatta M.S., Shokfai T., Nagarkatti M., Decho A.W., Tang C. (2018). Macromolecular-clustered facial amphiphilic antimicrobials. Nat. Commun..

[11] Nguyen L.T., Haney E.F., Vogel H.J. (2011). The expanding scope of antimicrobial peptide structures and their modes of action. Trends Biotechnol..

[12] Li J., Koh J.-J., Liu S., Lakshminarayanan R., Verma C.S., Beuerman R.W. (2017). Membrane active antimicrobial peptides: Translating mechanistic insights to design. Front. Neurosci..

[13] Costa F., Teixeira C., Gomes P., Martins M.C.L. (2019). Clinical application of AMPs. Adv. Exp. Med. Biol..

[14] Luo X., Song Y., Cao Z., Qin Z., Dessie W., He N., Wang Z., Tan Y. (2022). Evaluation of the antimicrobial activities and mechanisms of synthetic antimicrobial peptide against food-borne pathogens. Food Biosci..

[15] Ning Y., Han P., Ma J., Liu Y., Fu Y., Wang Z., Jia Y. (2021). Characterization of brevilaterins, multiple antimicrobial peptides simultaneously produced by *Brevibacillus laterosporus* S62-9, and their application in real food system. Food Biosci..

[16] Verma D.K., Thakur M., Singh S., Tripathy S., Gupta A.K., Baranwal D., Patel A.R., Shah N., Utama G.L., Niamah A.K. (2022). Bacteriocins as antimicrobial and preservative agents in food: Biosynthesis, separation and application. Food Biosci..

[17] Vishweshwaraiah Y.L., Acharya A., Hegde V., Prakash B. (2021). Rational design of hyperstable antibacterial peptides for food preservation. NPJ Sci. Food.

[18] Yang S., Li J., Aweya J.J., Yuan Z., Weng W., Zhang Y., Liu G.-M. (2020). Antimicrobial mechanism of *Larimichthys crocea* whey acidic protein-derived peptide (LCWAP) against *Staphylococcus aureus* and its application in milk. Int. J. Food Microbiol..

[19] Mora L., Reig M., Toldrá F. (2014). Bioactive peptides generated from meat industry by-products. Food Res. Int..

[20] Bersi G., Barberis S.E., Origone A.L., Adaro M.O. (2018). Bioactive peptides as functional food ingredients. Role of Materials Science in Food Bioengineering.

[21] Rivero-Pino F., Leon M.J., Millan-Linares M.C., Montserrat-de la Paz S. (2023). Antimicrobial plant-derived peptides obtained by enzymatic hydrolysis and fermentation as components to improve current food systems. Trends Food Sci. Technol..

[22] Arulrajah B., Muhialdin B.J., Zarei M., Hasan H., Saari N. (2020). Lacto-fermented Kenaf (*Hibiscus cannabinus* L.) seed protein as a source of bioactive peptides and their applications as natural preservatives. Food Control.

[23] Bi J., Tian C., Jiang J., Zhang G.-L., Hao H., Hou H.-M. (2020). Antibacterial activity and potential application in food packaging of peptides derived from turbot viscera hydrolysate. J. Agric. Food Chem..

[24] Moscoso-Mujica G., Zavaleta A.I., Mujica A., Arnao I., Moscoso-Neira C., Santos M., Sánchez J. (2021). Antimicrobial peptides purified from hydrolysates of kanihua (*Chenopodium pallidicaule* Aellen) seed protein fractions. Food Chem..

[25] Eckert E., Zambrowicz A., Pokora M., Setner B., Dąbrowska A., Szołtysik M., Szewczuk Z., Polanowski A., Trziszka T., Chrzanowska J. (2014). Egg-yolk protein by-product as a source of ACE-inhibitory peptides obtained with using unconventional proteinase from Asian pumpkin (*Cucurbita ficifolia*). J. Proteom..

[26] Pimchan T., Tian F., Thumanu K., Rodtong S., Yongsawatdigul J. (2023). Isolation, identification, and mode of action of antibacterial peptides derived from egg yolk hydrolysate. Poult. Sci..

[27] Zhao S., Qaiyumi S., Friedman S., Singh R., Foley S., White D., McDermott P., Donkar T., Bolin C., Munro S. (2003). Characterization of *Salmonella enterica* serotype Newport isolated from humans and food animals. J. Clin. Microbiol..

[28] Dennison S.R., Howe J., Morton L.H., Brandenburg K., Harris F., Phoenix D.A. (2006). Interactions of an anionic antimicrobial peptide with *Staphylococcus aureus* membranes. Biochem. Biophys. Res. Commun..

[29] Wang X., He L., Huang Z., Zhao Q., Fan J., Tian Y., Huang A. (2023). Isolation, identification and characterization of a novel antimicrobial peptide from *Moringa oleifera* seeds based on affinity adsorption. Food Chem..

[30] Zhao Q., Shi Y., Wang X., Huang A. (2020). Characterization of a novel antimicrobial peptide from buffalo casein hydrolysate based on live bacteria adsorption. J. Dairy Sci..

[31] Li S., Wang Y., Xue Z., Jia Y., Li R., He C., Chen H. (2021). The structure-mechanism relationship and mode of actions of antimicrobial peptides: A review. Trends Food Sci. Technol..

[32] Tkaczewska J. (2020). Peptides and protein hydrolysates as food preservatives and bioactive components of edible films and coatings-A review. Trends Food Sci. Technol..

[33] Chen Y., Guarnieri M.T., Vasil A.I., Vasil M.L., Mant C.T., Hodges R.S. (2007). Role of peptide hydrophobicity in the mechanism of action of α-helical antimicrobial peptides. Antimicrob. Agents Chemother..

[34] Tang Y.-L., Shi Y.-H., Zhao W., Hao G., Le G.-W. (2008). Insertion mode of a novel anionic antimicrobial peptide MDpep5 (Val-Glu-Ser-Trp-Val) from Chinese traditional edible larvae of housefly and its effect on surface potential of bacterial membrane. J. Pharm. Biomed. Anal..

[35] Almarwani B., Phambu N., Hamada Y.Z., Sunda-Meya A. (2020). Interactions of an anionic antimicrobial peptide with Zinc (II): Application to bacterial mimetic membranes. Langmuir.

[36] Brogden K.A. (2005). Antimicrobial peptides: Pore formers or metabolic inhibitors in bacteria?. Nat. Rev. Microbiol..

[37] Zhao Q., He L., Wang X., Ding X., Li L., Tian Y., Huang A. (2022). Characterization of a novel antimicrobial peptide isolated from *Moringa oleifera* seed protein hydrolysates and its membrane damaging effects on *Staphylococcus aureus*. J. Agric. Food Chem..

[38] Song R., Shi Q., Yang P., Wei R. (2017). Identification of antibacterial peptides from Maillard reaction products of half-fin anchovy hydrolysates/glucose via LC-ESI-QTOF-MS analysis. J. Funct. Foods..

[39] Torres M.D., Sothiselvam S., Lu T.K., de la Fuente-Nunez C. (2019). Peptide design principles for antimicrobial applications. J. Mol. Biol..

[40] Souza P.F., Marques L.S., Oliveira J.T., Lima P.G., Dias L.P., Neto N.A., Lopes F.E., Sousa J.S., Silva A.F., Caneiro R.F. (2020). Synthetic antimicrobial peptides: From choice of the best sequences to action mechanisms. Biochimie.

[41] Baltutis V., O’Leary P.D., Martin L.L. (2022). Self-assembly of linear, natural antimicrobial peptides: An evolutionary perspective. ChemPlusChem.

[42] Nielsen J.E., Bjørnestad V.A., Lund R. (2018). Resolving the structural interactions between antimicrobial peptides and lipid membranes using small-angle scattering methods: The case of indolicidin. Soft Matter.

[43] Zhou L., Lian K., Wang M., Jing X., Zhang Y., Cao J. (2022). The antimicrobial effect of a novel peptide LL-1 on *Escherichia coli* by increasing membrane permeability. BMC Microbiol..

[44] Rosenberg M., Azevedo N.F., Ivask A. (2019). Propidium iodide staining underestimates viability of adherent bacterial cells. Sci. Rep..

[45] Song W., Kong X., Hua Y., Chen Y., Zhang C., Chen Y. (2020). Identification of antibacterial peptides generated from enzymatic hydrolysis of cottonseed proteins. LWT.

[46] Hou J., Liu Z., Cao S., Wang H., Jiang C., Hussain M.A., Pang S. (2018). Broad-Spectrum antimicrobial activity and low cytotoxicity against human cells of a peptide derived from bovine αS1-casein. Molecules.

[47] Tian F., Rodtong S., Thumanu K., Hua Y., Roytrakul S., Yongsawatdigul J. (2022). Molecular insights into the mode of action of antibacterial peptides derived from chicken plasma hydrolysates. Foods.

[48] Harris F., Dennison S.R., Phoenix D.A. (2009). Anionic antimicrobial peptides from eukaryotic organisms. Curr. Protein Pept. Sci..

[49] Naumann D., Meyers R. (2000). Infrared spectroscopy in microbiology. Encyclopedia of Analytical Chemistry.

[50] Xiao J., Zhang H., Niu L., Wang X. (2011). Efficient screening of a novel antimicrobial peptide from *Jatropha curcas* by cell membrane affinity chromatography. J. Agric. Food Chem..

[51] Che Q., Zhou Y., Yang H., Li J., Xu X., Lai R. (2008). A novel antimicrobial peptide from amphibian skin secretions of *Odorrana grahami*. Peptides.

[52] Sun Y., Chang R., Li Q., Li B. (2016). Isolation and characterization of an antibacterial peptide from protein hydrolysates of *Spirulina platensis*. Eur. Food Res. Technol..

[53] Nakajima Y., Ishibashi J., Yukuhiro F., Asaoka A., Taylor D., Yamakawa M. (2003). Antibacterial activity and mechanism of action of tick defensin against Gram-positive bacteria. Biochim. Biophys. Acta Gen. Subj..

[54] Liu Y., Xia X., Xu L., Wang Y. (2013). Design of hybrid β-hairpin peptides with enhanced cell specificity and potent anti-inflammatory activity. Biomaterials.

[55] Liao H., Zhang F., Liao X., Hu X., Chen Y., Deng L. (2010). Analysis of *Escherichia coli* cell damage induced by HPCD using microscopies and fluorescent staining. Int. J. Food Microbiol..

[56] Stark M., Liu L.-P., Deber C.M. (2002). Cationic hydrophobic peptides with antimicrobial activity. Antimicrob. Agents Chemother..

